# Characteristics of Very High-Power, Short-Duration Radiofrequency Applications

**DOI:** 10.3389/fcvm.2022.941434

**Published:** 2022-07-13

**Authors:** Gábor Orbán, Zoltán Salló, Péter Perge, Pál Ábrahám, Katalin Piros, Klaudia Vivien Nagy, István Osztheimer, Béla Merkely, László Gellér, Nándor Szegedi

**Affiliations:** Heart and Vascular Center, Semmelweis University, Budapest, Hungary

**Keywords:** atrial fibrillation, ablation, very high power, short duration, pulmonary vein isolation

## Abstract

**Introduction:**

Pulmonary vein isolation is the cornerstone of rhythm-control therapy for atrial fibrillation (AF). The very high-power, short-duration (vHPSD) radiofrequency (RF) ablation is a novel technology that favors resistive heating while decreasing the role of conductive heating. Our study aimed to evaluate the correlations between contact force (CF), power, impedance drop (ID), and temperature; and to assess their role in lesion formation with the vHPSD technique.

**Methods:**

Consecutive patients who underwent initial point-by-point RF catheter ablation for AF were enrolled in the study. The vHPSD ablation was performed applying 90 W for 4 s with an 8 ml/min irrigation rate.

**Results:**

Data from 85 patients [median age 65 (59–71) years, 34% female] were collected. The median procedure time, left atrial dwelling time, and fluoroscopy time were 70 (60–90) min, 49 (42–58) min, and 7 (5–11) min, respectively. The median RF time was 312 (237-365) sec. No steam pop nor major complications occurred. A total of 6,551 vHPSD RF points were analyzed. The median of CF, maximum temperature, and ID were 14 (10–21) g, 47.6 (45.1–50.4) °C, and 8 (6–10) Ohms, respectively. CF correlated significantly with the maximum temperature (*p* < 0.0001). A CF of 5 g and above was associated with a significantly higher temperature compared to those lesions with a CF below 5 grams (*p* < 0.0001). Bilateral first-pass isolation rate was 84%. The 6-month AF-recurrence rate was 7%.

**Conclusion:**

The maximum temperature and CF significantly correlate with each other during vHPSD applications. A CF ≥ 5 g leads to better tissue heating and thus might be more likely to result in good lesion formation, although this clinical study was unable to assess actual lesion sizes.

## Introduction

Atrial fibrillation (AF) is the most common sustained cardiac arrhythmia worldwide (1). Pulmonary vein isolation (PVI) is the most effective treatment for AF (2). However, the success rate of catheter ablation shows great variability (3, 4). Therefore, several predictors of AF recurrence have been described, such as hypertension, left atrial enlargement, and certain anatomical variations of pulmonary veins (PVs) and the left atrial ridge (5–8). In addition, numerous technological innovations in catheter ablation are also being sought to reduce recurrence, including the CLOSE protocol and the measurement of local impedance (9–14). Still, AF recurrence remained a critical issue (15, 16). The QDOT Micro ablation catheter (Biosense Webster, Inc., Irvine, CA, USA) is a novel contact force-sensing catheter developed for very high-power, short-duration (vHPSD) temperature-controlled radiofrequency (RF) ablation. In such cases, ablation is applied with 90 W for 4 s. The aim of vHPSD ablation is to favor resistive heating while decreasing the role of conductive heating (17). Thus, lesion geometry will be different compared to conventional low-power long-duration ablation. Still, the shallower lesions are thought to be transmural in the left atrium (LA) (18, 19). The use of vHPSD ablation shows remarkable initial results (20, 21). However, limited data are available regarding lesion creation with this novel technology. Understanding the details of vHPSD ablation are essential as we apply such a high energy (i.e., 90 W) for the first time in clinical practice. The necessity of analyzing the vHPSD lesion creation is also underlined by the fact that vHPSD ablation works in an innovative, quickly regulated temperature-controlled mode, as opposed to the power-controlled mode of traditional RF ablation techniques. Our study aimed to evaluate the correlations between contact force (CF), power, impedance drop (ID), and temperature; and to assess their role in lesion formation with the vHPSD technique.

## Methods

### Patient Population

Consecutive patients were enrolled in our prospective study undergoing first RF catheter ablation for AF at the Heart and Vascular Center of Semmelweis University, Budapest, Hungary. All patients agreed to the ablation procedure and provided written informed consent to data retrieval and analysis. Ethics approval was waived by Semmelweis University Regional and Institutional Committee of Science and Research Ethics (No.: 278/2020.) and was in accordance with the declarations of Helsinki.

### Catheter Ablation Procedure

**Figure 1:**
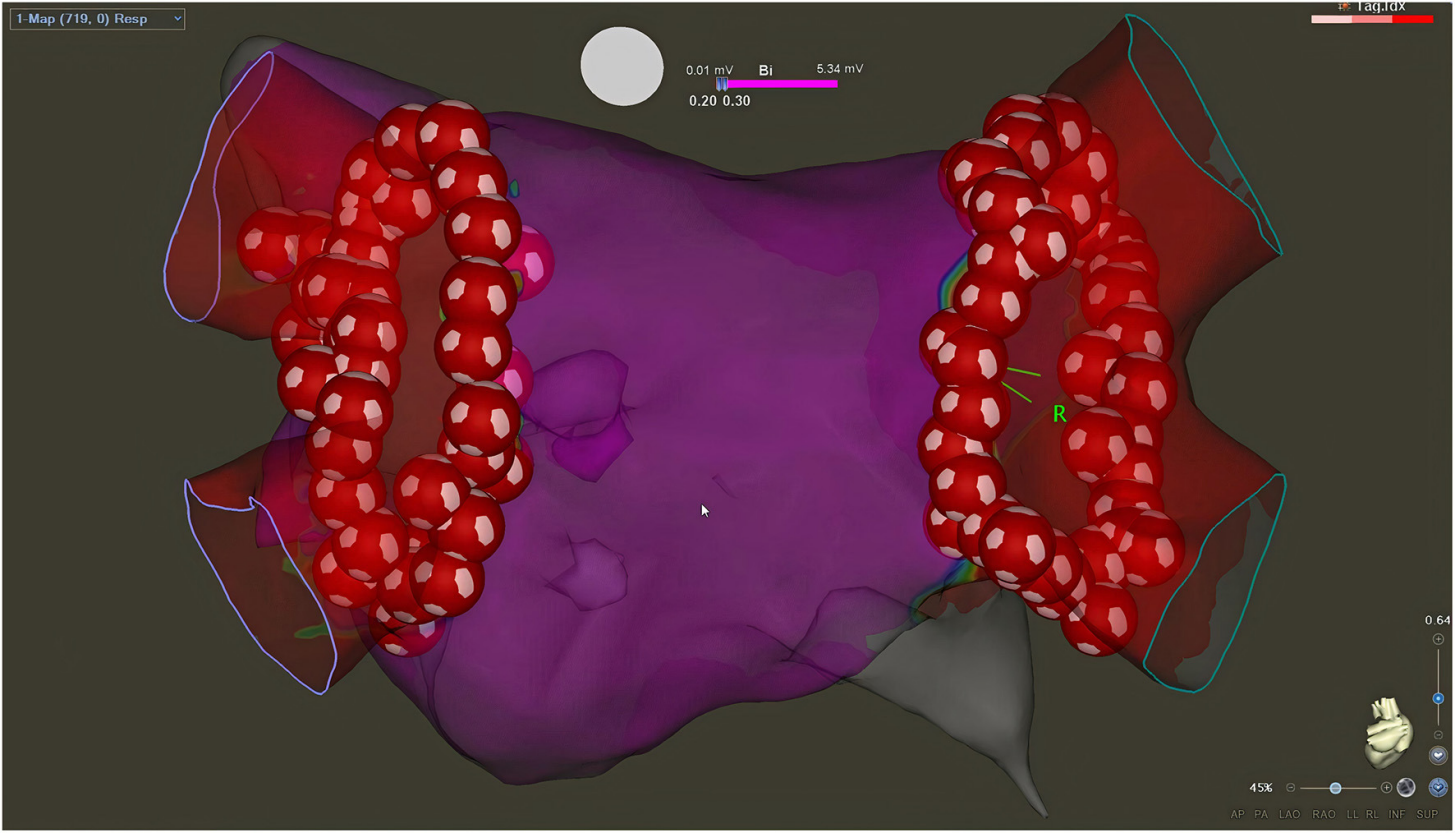
Left atrial voltage map created with CARTO3 electroanatomical mapping system after successful PVI with vHPSD ablation (postero-anterior view). The inter-tag distances are <5 mm between all neighboring points (Heart and Vascular Center, Semmelweis University).

**Figure 2:**
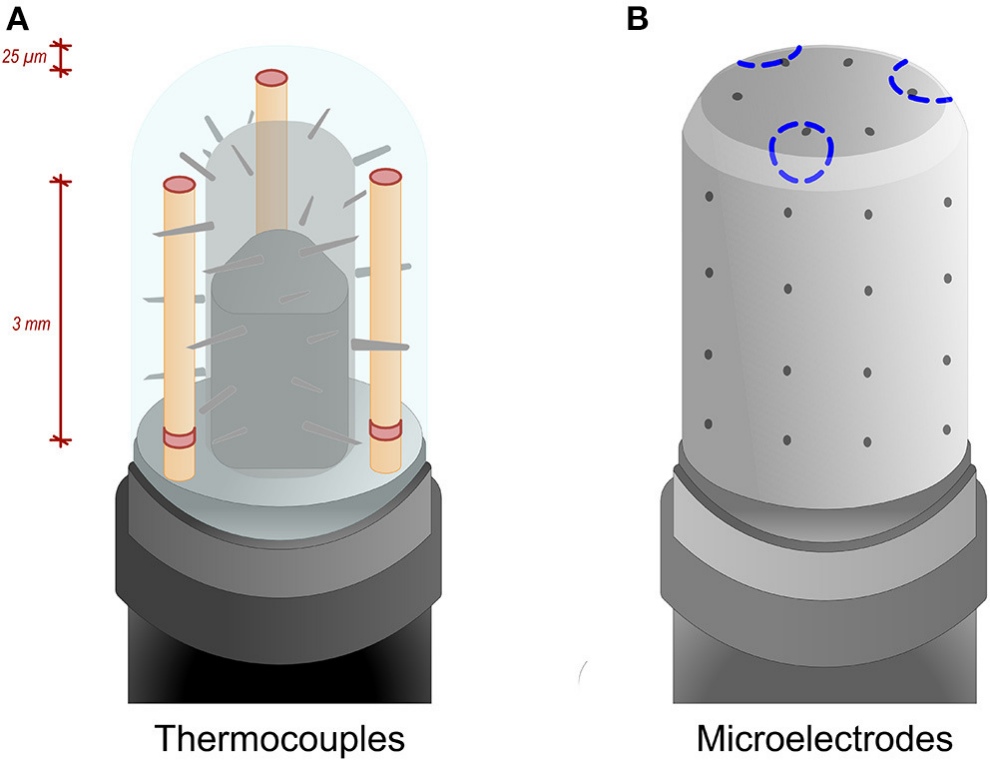
**(A,B)** QDOT Micro ablation catheter illustration. **(A)** The ablation catheter incorporates six thermocouples (columns with red disk) symmetrically embedded in the tip for the purpose of highly accurate local temperature measurement. The three distal thermocouples are positioned at a distance of 25 μm from the tip, while the proximal ones are positioned 3 mm proximally. **(B)** Three microelectrodes (blue dotted circles) are also located in the catheter tip. Each has a surface area of 0.17 mm^2^ and an interelectrode spacing of 1.5 mm.

**Figure 3:**
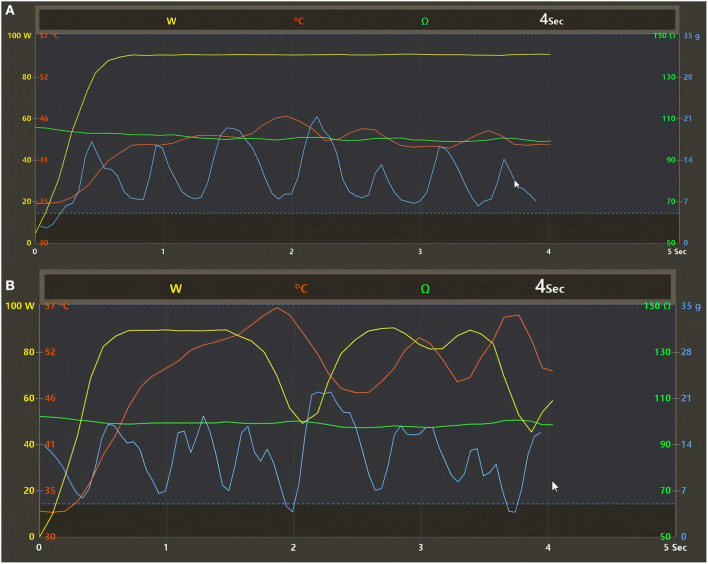
**(A,B)** Regulation of the temperature control mode with the QDOT irrigated catheter and vHPSD algorithm as registered by the CARTO3 electroanatomical mapping system (Heart and Vascular Center, Semmelweis University). **(A)** The 90 W of power is stably delivered throughout the 4 s when the temperature registered by any thermocouples located distally around the catheter tip does not reach the target temperature (55°C) during lesion formation. **(B)** When the target temperature is reached, the ablation power is immediately downregulated by the vHPSD algorithm to prevent overheating of the tissue. Yellow line, delivered power; red line, temperature; blue line, contact force; green line, impedance.

Procedures were performed under conscious sedation and by experienced operators (>100 AF ablations/year). Femoral venous access was used for all procedures. Double transseptal puncture was conducted to access the LA under fluoroscopy guidance and pressure monitoring. First, a fast anatomical map of the LA was obtained with the CARTO mapping system (CARTO3, Biosense Webster, Inc., Irvine, CA, USA) and a multipolar mapping catheter (Lasso^®^ NAV Eco, Biosense Webster, Inc., Irvine, CA, USA, or PentaRay Nav Eco, Biosense Webster, Inc., Irvine, CA, USA). Subsequently, vHPSD ablation was performed using the QDOT Micro ablation catheter (Biosense Webster, Inc., Irvine, CA, USA) with Agilis NxT steerable introducer (Abbott). Point-by-point RF applications were delivered around the antra of the ipsilateral PVs to achieve complete electrical isolation of all PVs from the LA (Figure 1). Such vHPSD ablation was performed applying 90 W for 4 s with an 8 ml/min irrigation rate. Besides being capable of contact force-sensing, the QDOT Micro ablation catheter incorporates six thermocouples embedded in the catheter tip, used for highly accurate local temperature measurement covering the whole distal electrode. The three distal thermocouples are positioned at a distance of 25 μm from the tip, while the proximal ones are positioned 3 mm proximally (Figures 2A,B). Accurate and reliable temperature measurement at the tip-tissue interface was not previously possible due to the interfering effect of the cold irrigation fluid used during ablation. To overcome this limitation, an algorithm was developed and then validated to determine the actual tip-tissue interface temperature based on the temperature measured by the thermocouples. The arrangement of electrodes is optimized to record the temperature at both perpendicular and parallel catheter orientations; thus providing the basis of a susceptible feedback system of catheter-tissue interface temperature and thereby catheter stability during ablation (17, 20). When the operator starts the RF application, a 2-s period with an irrigation rate of 8 ml/min begins to cool the tissue surface prior to high power RF delivery. Following this 2-s delay, RF delivery is started, and the power rapidly increases up to 90 W. During RF delivery, the vHPSD algorithm continuously modulates the power based on the hottest surface temperature measured by the thermocouples: the target temperature is set at 55°C, the cut-off temperature is set at 65°C. The 90 W of power is stably delivered throughout the 4 s if the temperature registered by any thermocouples does not reach the target temperature (Figure 3A). If the target temperature is reached, the ablation power is downregulated to prevent overheating of the tissue (Figure 3B). If the cut-off temperature is reached, the RF delivery is immediately stopped. After each RF application, the 8 ml/min irrigation continues for 4 s. The ablation points are registered automatically (Carto VISITAG Module, Biosense Webster, Inc., Irvine, CA, USA). We applied overlapping ablation points; thus, the inter-tag distances were <5 mm between all neighboring points. No additional ablations were performed beyond the pulmonary vein isolation. PV disconnection was carefully examined in all cases, as both entrance and exit blocks were verified. First-pass isolation was defined as the presence of both entrance and exit block after completion of the first-pass circumferential ablation around the antra of the ipsilateral PVs. In the absence of disconnection after completing the circumferential ablation, mapping catheter-guided additional vHPSD ablation was delivered until complete isolation was achieved. All patients without complications were discharged the day after the procedure.

### Follow-Up and Definition of Recurrence

After the procedure, outpatient clinical follow-up visits were scheduled at 3 and 6 months. Whenever patients experienced symptoms of arrhythmia, additional visits were scheduled. Follow-up visits included clinical assessment of the patients and 24 h Holter ECG monitoring. Recurrence of AF was defined as the occurrence of atrial tachyarrhythmia that lasts for more than 30 s, documented by ECG.

### Data Collection

The following data of each vHPSD application was collected: mean delivered power, temperature change, maximum temperature, generator impedance drop (ID), mean contact force (CF), minimum CF, maximum CF. All registered VISITAG points were exported from the system for offline analysis. Hereinafter, all reported CF values indicate the mean CF values unless otherwise stated.

### Statistical Analysis

Most of the variables showed non-parametric distributions after performing the Shapiro-Wilk test. Thus, the continuous variables were expressed as medians and interquartile ranges. Continuous variables were compared with the Mann-Whitney test. The Spearman's ρ correlation coefficient was used to assess the correlation between variables. Statistical analyses were performed using IBM SPSS 25 (Apache Software Foundation, USA) and GraphPad Prism 9.1.2 (GraphPad Softwares Inc., USA) software products.

## Results

### Characteristics of the Study Population and Ablation Points, Procedural Outcomes

Data of 85 consecutive patients [median age 65 (59–71) years, 34% female] were collected. The median BMI was 29 (26–31) kg/m^2^, and 75% of them had hypertension. Eighteen percent had diabetes, and 21% of them had hyperlipidemia. The median of procedure time, left atrial dwelling time, and fluoroscopy time were 70 (60–90) min, 49 (42–58) min, and 7 (5–11) min, respectively. The median RF time was 312 (237–365) s. Baseline characteristics of the study population and the procedures are shown in Table 1. A total of 6,551 vHPSD RF points were analyzed. The median of CF, maximum temperature, and ID were 14 (10–21) g, 47.6 (45.1–50.4) °C, and 8 (6–10) Ohms, respectively. Detailed parameters of the lesions are presented in Table 2. Bilateral first-pass isolation rate was 84%. The rate of AF-recurrence 6 months after ablation was 7%. No steam pop nor major complications occurred. There was one case of groin hematoma that did not require intervention.

**Table 1:** Baseline characteristics of the study population and the procedures.

**Patient characteristics (*n* = 85)**
Age (years)	65 (59–71)
Female, *n* (%)	29 (34)
AF type
Paroxysmal, *n* (%)	51 (60)
Persistent, *n* (%)	34 (40)
BMI (kg/m^2^)	29 (26–31)
Hypertension, *n* (%)	64 (75)
Hyperlipidemia, *n* (%)	18 (21)
Diabetes, *n* (%)	15 (18)
Prior stroke/TIA, *n* (%)	4 (5)
CAD, *n* (%)	16 (19)
Thyroid gland disease, *n* (%)	6 (7)
CHA_2_DS_2_-VASc score	3 (1–4)
**Procedure characteristics (*****n*** **=** **85)**
Procedure time (min)	70 (60–90)
LA dwelling time (min)	49 (42–58)
Fluoroscopy time (min)	7 (5–11)
RF time (s)	312 (237–365)

*AF, atrial fibrillation; BMI, body mass index; CAD, coronary artery disease; LA, left atrium; RF, radiofrequency; TIA, transient ischemic attack. Continuous variables are expressed as medians and interquartile ranges*.

**Table 2:** Baseline characteristics of vHPSD lesions, *n* = 6,551.

Mean delivered power (W)	84 (84–86)
Maximum temperature (°C)	47.6 (45.1–50.4)
Temperature change (°C)	13 (10.6–15.8)
Generator impedance drop (Ohm)	8 (6–10)
Contact force (g)	14 (10–21)
Minimum contact force (g)	6 (3–11)
Maximum contact force (g)	24 (17–35)

*Variables are expressed as medians and interquartile ranges*.

### Correlations Between RF Application Parameters

**Figure 4:**
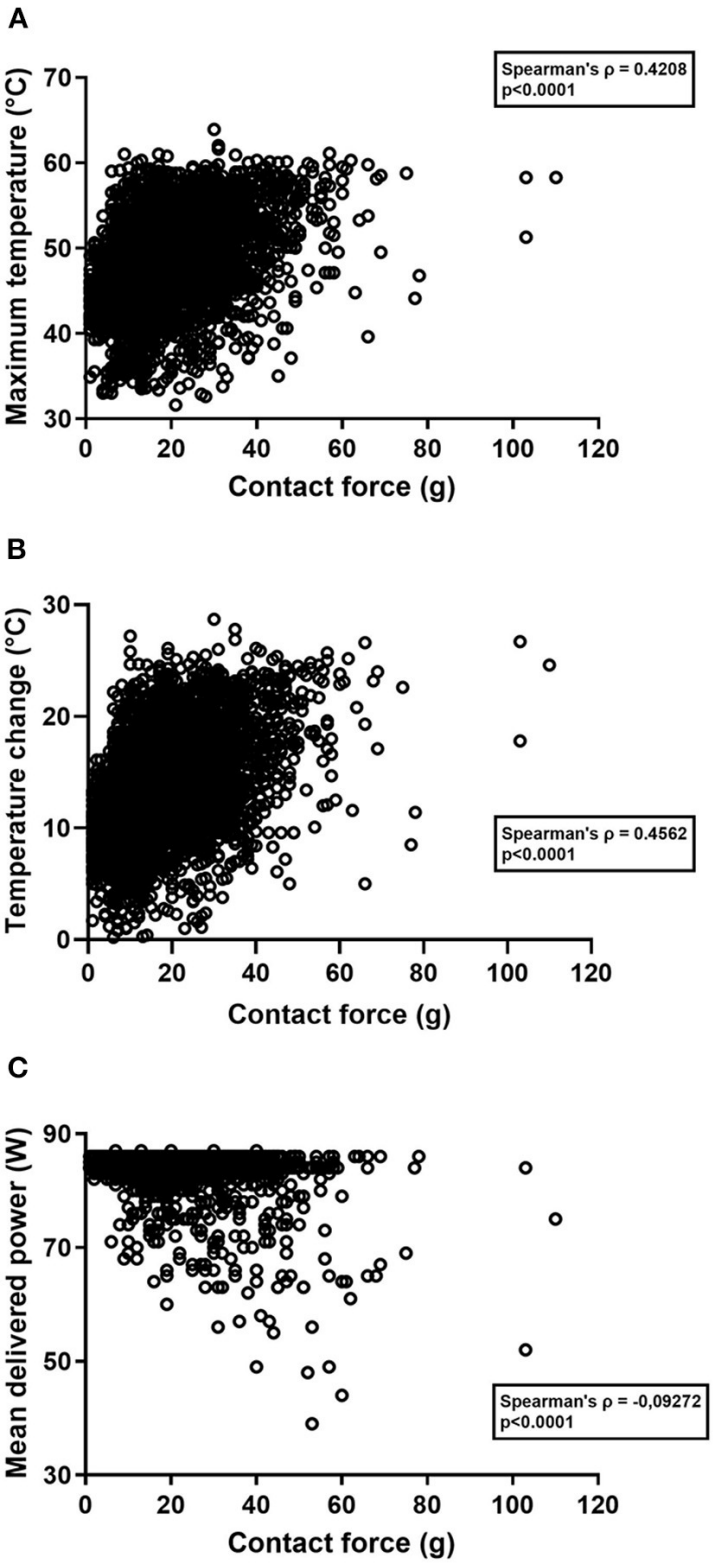
**(A–C)** Scatter plot diagrams of the correlations between different parameters registered during radiofrequency applications. **(A)** Correlation between contact force and the maximum temperature. **(B)** Correlation between contact force and temperature change. **(C)** Correlation between contact force and the mean delivered power. ρ, correlation coefficient.

CF correlated significantly with the maximum temperature (Spearman's ρ = 0.4208, *p* < 0.0001) (Figure 4A), and with temperature change (Spearman's ρ = 0.4562, *p* < 0.0001) (Figure 4B). There was a negative correlation between CF and the mean delivered power (Spearman's ρ = −0.09272, *p* < 0.0001) (Figure 4C).

There was also a significant correlation between ID and the maximum temperature (Spearman's ρ = 0.373, *p* < 0.0001) (Supplementary Figure 1A), ID and the mean delivered power (Spearman's ρ = −0.07184, *p* < 0.0001) (Supplementary Figure 1B), and ID and temperature change (Spearman's ρ = 0.3822, *p* < 0.0001) (Supplementary Figure 1C). ID showed a significant correlation with CF (Spearman's ρ = 0.1907, *p* < 0.0001) (Supplementary Figure 1D), too.

### Maximum Temperature of Lesions Created With a CF Lower Than 5 g and Those With a CF of 5 g or More

**Figure 5:**
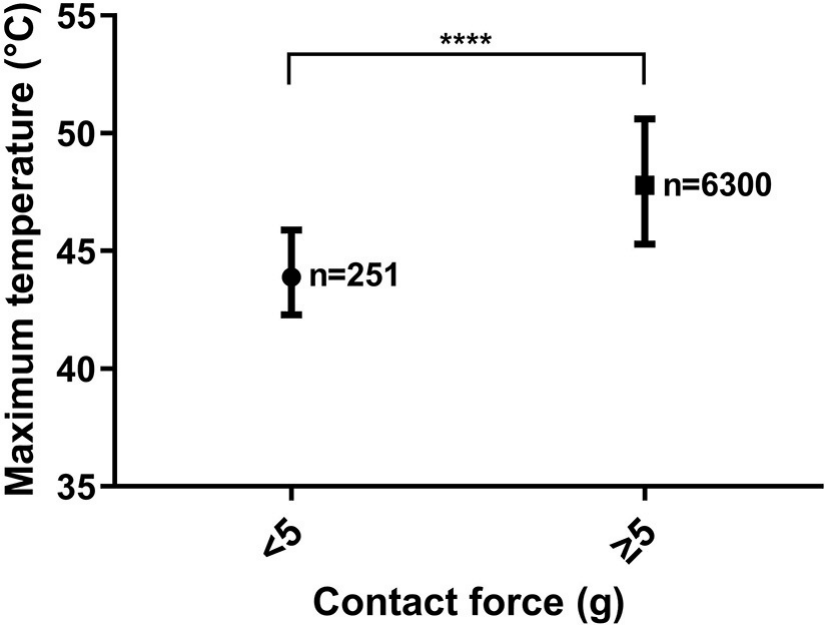
Difference in the maximum temperature between lesions created with a CF <5 g and those with a CF ≥ 5 g. **** = *p* < 0.0001. Median and interquartile ranges.

The maximum temperature registered by the thermocouples of the catheter was significantly higher in applications created with a CF of 5 grams and above compared to those with a CF below 5 grams [47.8 (45.3–50.6) and 43.9 (42.3–45.9) °C, *p* < 0.0001] (Figure 5). Further analysis of the parameters are shown in Supplementary Figures 2, 3.

## Discussion

### Main Findings

Our main findings are that the maximum temperature and CF significantly correlate with each other during vHPSD RF applications. Moreover, a CF ≥5 g is associated with significantly higher maximum temperatures during RF delivery.

### Parameters Used for Assessing Efficacy and Safety of Ablation Lesions

Recently, numerous new parameters have been identified to assess the quality of the ablation lesions in atrial fibrillation ablation, such as ablation index, lesion size index, and changes in local impedance (11, 22, 23). These all provide valuable information regarding proper lesion formation. However, the evaluation of the abovementioned factors is not technically available during vHPSD applications as both time and power are set to a fixed value in the case of vHPSD ablation. In addition, the QDOT Micro ablation catheter is also unable to measure changes in local impedance. Nevertheless, the change in generator impedance is measured by the system. Previously, several studies have examined the ablation-induced drop in generator impedance as an indicator of lesion formation (24, 25). However, it was clearly showed recently that changes in generator impedance show a weak correlation with lesion formation (26).

Gutbrod et al. (13) showed that the local impedance of the tissue is significantly higher when CF is at least 5 g compared to those with an applied CF below 5 g. However, when comparing different CF values above 5 g, they found no significant difference in local impedance. This suggests that once CF reaches a value of 5 g, stable contact with the tissue is achieved, and a further increase in CF does not result in significantly greater stability (13). Consistent with this, we also found that a CF ≥ 5 g results in significantly higher maximum temperatures during ablation (Figure 5). Higher maximum temperatures measured by the electrode correlate with higher tissue heating, which determines the size of the resistive heating zone (18). Therefore, higher maximum electrode temperatures during vHPSD ablation correlate with larger resistive heating zones and a CF ≥ 5 g results in more extensive resistive heating during lesion formation.

### Regulation of the Temperature Control Mode With the QDOT Irrigated Catheter

Previously available open-irrigated catheter technologies did not provide adequate temperature feedback to use temperature control mode for power delivery, as the temperature measured by the thermocouples was not appropriate, due to the proximal location of the temperature sensors. Previously, irrigated catheters could only be used in power control mode, which delivers a consistent set of power without being affected by measured temperatures, other than to terminate RF application if the temperature is too high.

The technology in the QDOT Micro Catheter enables temperature feedback with open irrigation. The temperature feedback allowed by this novel technology enables a unique temperature control mode of radiofrequency energy delivery (Figures 3A,B). Three thermocouples are located distally, 25 μm from the catheter tip, and three thermocouples are located 3 mm proximally (Figure 2A). This design places the thermocouples in close proximity to the tip-tissue interface, enabling the determination of accurate surface temperatures during RF energy delivery based on the temperature measured by the thermocouples.

**Figure 6:**
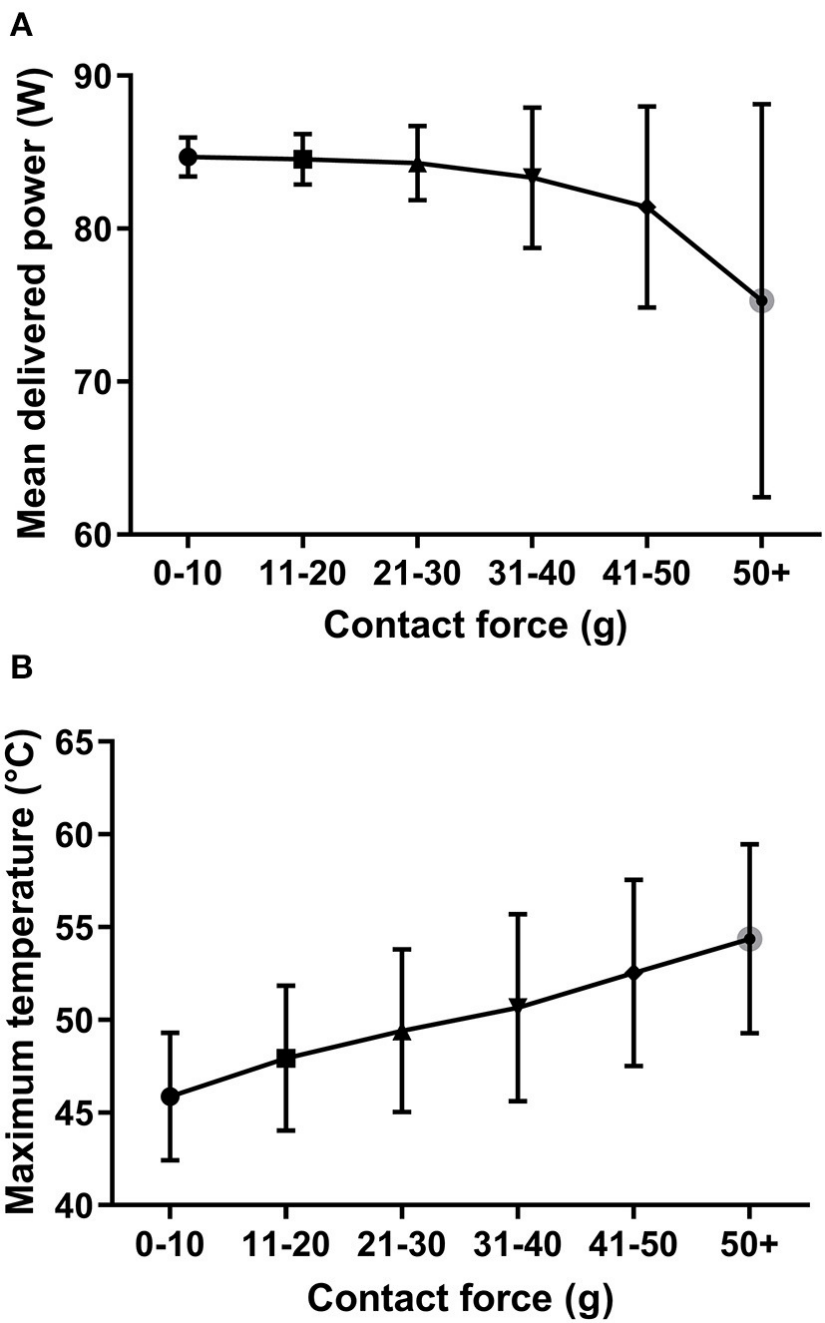
**(A,B)** The association of the mean delivered power and maximum temperature to the different contact force ranges. Increasing contact force values result in higher temperatures but a lower delivered energy. This is the consequence of the appropriate regulation of the ablation generator. Median and interquartile ranges.

At higher CFs, the maximum temperature might increase quickly and reach the target temperature level (Figures 3B, 6B). In such cases, the algorithm reduces the amount of power delivered to prevent overheating of the tissues (20). Based on our data, Figure 6A shows that there is a tendency for a decreased mean delivered power at higher CF values.

On the other hand, down-regulation of the power does not seem to have a negative influence on lesion creation, as target temperature will be reached in such cases, resulting in an optimal resistively heated area (Figure 6B). Overall, an increasing CF value will result in higher temperatures (and higher ID) but lower delivered energy due to the appropriate and quick regulation of the ablation generator (Figures 6A,B; Supplementary Figure 4).

### Role of Contact Force Values in vHPSD AF Ablation

Catheter stability with adequate CF is critical for optimal lesion formation. However, too high CF carries a significantly increased risk of steam pop and cardiac perforation (27). Neuzil et al. (28) showed a target CF of 20 g as the optimal value regarding new lesion formation, with a recommended range of 10–30 g. In a subsequent study, Kautzner et al. (29) further confirmed that keeping CF at such target values leads to more durable lesion formation during PVI. However, the issue of optimal CF for vHPSD ablation has not been extensively studied in the past. In our present work, we have concluded that a CF of 5 g and above leads to optimal tissue heating, which confirms a slightly lower CF range than the previously used clinical practice based on the EFFICAS I and II studies (28, 29).

The manufacturer's recommended CF range when using the QDOT Micro ablation catheter is 5–25 g. In our current real-world study, higher CF values also occurred (e.g., 50 g), which did not lead to steam pops or cardiac perforations, most likely due to the quick real-time regulation of the applications' power according to the actual temperature. Higher than recommended CF values might occur due to the procedures being performed under conscious sedation when sudden changes in the breathing pattern or slight movements of the patient could result in high CF values (30). However, we assume that at higher than recommended CF values, the target temperature is reached sooner, which is compensated by the generator by reducing the energy delivery; thus reducing the possibility of steam pops (Figure 6B). Moreover, we have shown that a CF ≥ 5 g results in significantly higher electrode temperature, thus, tissue temperature will also be higher, yielding a larger resistive heating zone.

### Limitations

There are limitations to our work. It was a single-center, single-arm study with relatively low number of patients involved. Nevertheless, the number of RF applications investigated was high enough to yield statistically significant results. Given the clinical nature of the current study, actual lesion sizes could not be assessed. As only mid-term clinical follow-up is reported, the impact of the observed associations on the long-term clinical success is unknown. Thus, further investigation regarding the long-term clinical value of our findings will be necessary in the future. Moreover, the study population is low to draw conclusions regarding the procedure's safety profile. We also mention the fact that vHPSD ablation is currently only possible with one proprietary catheter compatible only with the electrophysiology system of the same vendor.

## Conclusion

The maximum temperature and CF significantly correlate with each other during vHPSD applications. A CF ≥ 5 g leads to better tissue heating and thus might be more likely to result in good lesion formation in the LA, although this clinical study was unable to assess actual lesion sizes.

## Data Availability Statement

The raw data supporting the conclusions of this article will be made available by the authors, without undue reservation.

## Ethics Statement

The studies involving human participants were reviewed and approved by Semmelweis University Regional and Institutional Committee of Science and Research Ethics (SE RKEB No.: 278/2020.). The patients/participants provided their written informed consent to participate in this study.

## Author Contributions

GO made the basic conception, collected and analyzed the data, and wrote the manuscript. PP helped analyzing the data. ZS, KP, PÁ, KN, and IO revised the article. BM revised and approved the manuscript to be published. LG and NS helped in the conception phase, made critical revision of the article, and approved the final version to be published. All authors contributed to the article and approved the submitted version.

## Funding

This study was supported by the National Research, Development and Innovation Office of Hungary (NKFIA; NVKP_16-1-2016-0017 National Heart Program) and by the MD-PhD Excellence Program of Semmelweis University (EFOP-3.6.3-VEKOP-16-2017-00009). The research was financed by the Thematic Excellence Programme (Tématerületi Kiválósági Program, 2020-4.1.1.-TKP2020) of the Ministry for Innovation and Technology in Hungary, within the framework of the Therapeutic Development and Bioimaging programmes of the Semmelweis University.

## Conflict of Interest

NS, ZS, and LG report consulting fees from Biosense Webster, not related to the present work. The remaining authors declare that the research was conducted in the absence of any commercial or financial relationships that could be construed as a potential conflict of interest.

## Publisher's Note

All claims expressed in this article are solely those of the authors and do not necessarily represent those of their affiliated organizations, or those of the publisher, the editors and the reviewers. Any product that may be evaluated in this article, or claim that may be made by its manufacturer, is not guaranteed or endorsed by the publisher.

## Abbreviations

AF: Atrial Fibrillation
PVI: Pulmonary Vein Isolation
PV: Pulmonary Vein
vHPSD: Very High-Power Short-Duration
RF: Radiofrequency
LA: Left Atrium
CF: Contact Force
ID: Impedance Drop.

## References

[1] Hindricks G Potpara T Dagres N Arbelo E Bax JJ Blomstrom-Lundqvist C. 2020 ESC Guidelines for the diagnosis and management of atrial fibrillation developed in collaboration with the European association for cardio-thoracic surgery (EACTS). Eur Heart J. (2021) 42:373–498. 10.1093/eurheartj/ehaa612 32860505

[2] Calkins H Hindricks G Cappato R Kim YH Saad EB Aguinaga L. 2017 HRS/EHRA/ECAS/APHRS/SOLAECE expert consensus statement on catheter and surgical ablation of atrial fibrillation. Europace. (2018) 20:e1–60. 10.1093/europace/eux274 29016840 PMC5834122

[3] Nielsen JC Johannessen A Raatikainen P Hindricks G Walfridsson H Pehrson SM. Long-term efficacy of catheter ablation as first-line therapy for paroxysmal atrial fibrillation: 5-year outcome in a randomised clinical trial. Heart. (2017) 103:368–76. 10.1136/heartjnl-2016-309781 27566295

[4] Arbelo E Brugada J Blomström-Lundqvist C Laroche C Kautzner J Pokushalov E. Contemporary management of patients undergoing atrial fibrillation ablation: in-hospital and 1-year follow-up findings from the ESC-EHRA atrial fibrillation ablation long-term registry. Eur Heart J. (2017) 38:1303–16. 10.1093/eurheartj/ehw564 28104790

[5] Szegedi N Vecsey-Nagy M Simon J Szilveszter B Herczeg S Kolossváry M. Orientation of the right superior pulmonary vein affects outcome after pulmonary vein isolation. Eur Heart J Cardiovasc Imaging. (2022) 23:515–23. 10.1093/ehjci/jeab111 33693618

[6] Simon J El Mahdiui M Smit JM Száraz L van Rosendael AR Herczeg S. Left atrial appendage size is a marker of atrial fibrillation recurrence after radiofrequency catheter ablation in patients with persistent atrial fibrillation. Clin Cardiol. (2022) 45:273–81. 10.22541/au.162532621.15263837/v1 34799870 PMC8922535

[7] El Mahdiui M Simon J Smit JM Kuneman JH van Rosendael AR Steyerberg EW. Posterior left atrial adipose tissue attenuation assessed by computed tomography and recurrence of atrial fibrillation after catheter ablation. Circ Arrhythm Electrophysiol. (2021) 14:e009135. 10.1161/CIRCEP.120.009135 33720759

[8] Szegedi N Simon J Szilveszter B Salló Z Herczeg S Száraz L. Abutting left atrial appendage and left superior pulmonary vein predicts recurrence of atrial fibrillation after point-by-point pulmonary vein isolation. Front Cardiovasc Med. (2022) 9:708298. 10.3389/fcvm.2022.708298 35242821 PMC8885731

[9] Taghji P El Haddad M Phlips T Wolf M Knecht S Vandekerckhove Y. Evaluation of a strategy aiming to enclose the pulmonary veins with contiguous and optimized radiofrequency lesions in paroxysmal atrial fibrillation: a pilot study. JACC Clin Electrophysiol. (2018) 4:99–108. 10.1016/j.jacep.2017.06.023 29600792

[10] Phlips T Taghji P El Haddad M Wolf M Knecht S Vandekerckhove Y. Improving procedural and one-year outcome after contact force-guided pulmonary vein isolation: the role of interlesion distance, ablation index, and contact force variability in the 'CLOSE'-protocol. Europace. (2018) 20:f419–27. 10.1093/europace/eux376 29315411

[11] Szegedi N Salló Z Perge P Piros K Nagy VK Osztheimer I. The role of local impedance drop in the acute lesion efficacy during pulmonary vein isolation performed with a new contact force sensing catheter-a pilot study. PLoS ONE. (2021) 16:e0257050. 10.1371/journal.pone.0257050 34529678 PMC8445471

[12] Park JW Yang SY Kim M Yu HT Kim TH Uhm JS. Efficacy and safety of high-power short-duration radiofrequency catheter ablation of atrial fibrillation. Front Cardiovasc Med. (2021) 8:709585. 10.3389/fcvm.2021.709585 34692779 PMC8530188

[13] Gutbrod SR Shuros A Koya V Alexander-Curtis M Lehn L Miklos K. Improved ablation efficiency in pvi guided by contact force and local impedance: chronic canine model. Front Physiol. (2021) 12:808541. 10.3389/fphys.2021.808541 35082695 PMC8784686

[14] Mao ZJ Pei Y Lin H Xiang Y Huang ZQ Xiao FY. Assessment of high-power catheter ablation in patients with atrial fibrillation: a meta-analysis. Front Cardiovasc Med. (2021) 8:609590. 10.3389/fcvm.2021.609590 34746245 PMC8564349

[15] De Pooter J Strisciuglio T El Haddad M Wolf M Phlips T Vandekerckhove Y. Pulmonary vein reconnection no longer occurs in the majority of patients after a single pulmonary vein isolation procedure. JACC Clin Electrophysiol. (2019) 5:295–305. 10.1016/j.jacep.2018.11.020 30898231

[16] Li Z Wang S Hidru TH Sun Y Gao L Yang X. Long atrial fibrillation duration and early recurrence are reliable predictors of late recurrence after radiofrequency catheter ablation. Front Cardiovasc Med. (2022) 9:864417. 10.3389/fcvm.2022.864417 35402564 PMC8990906

[17] Barkagan M Contreras-Valdes FM Leshem E Buxton AE Nakagawa H Anter E. High-power and short-duration ablation for pulmonary vein isolation: Safety, efficacy, and long-term durability. J Cardiovasc Electrophysiol. (2018) 29:1287–96. 10.1111/jce.13651 29846987

[18] Nakagawa H Ikeda A Sharma T Govari A Ashton J Maffre J. Comparison of in vivo tissue temperature profile and lesion geometry for radiofrequency ablation with high power-short duration and moderate power-moderate duration: effects of thermal latency and contact force on lesion formation. Circ Arrhythm Electrophysiol. (2021) 14:e009899. 10.1161/CIRCEP.121.009899 34138641

[19] Piatek-Koziej K Hołda J Tyrak K Bolechała F Strona M Koziej M. Anatomy of the left atrial ridge (coumadin ridge) and possible clinical implications for cardiovascular imaging and invasive procedures. J Cardiovasc Electrophysiol. (2020) 31:220–6. 10.1111/jce.14307 31808228

[20] Leshem E Zilberman I Tschabrunn CM Barkagan M Contreras-Valdes FM Govari A. High-power and short-duration ablation for pulmonary vein isolation: biophysical characterization. JACC Clin Electrophysiol. (2018) 4:467–79. 10.1016/j.jacep.2017.11.018 30067486

[21] Reddy VY Grimaldi M De Potter T Vijgen JM Bulava A Duytschaever MF. Pulmonary vein isolation with very high power, short duration, temperature-controlled lesions: the QDOT-FAST trial. JACC Clin Electrophysiol. (2019) 5:778–86. 10.1016/j.jacep.2019.04.009 31320006

[22] Das M Loveday JJ Wynn GJ Gomes S Saeed Y Bonnett LJ. Ablation index, a novel marker of ablation lesion quality: prediction of pulmonary vein reconnection at repeat electrophysiology study and regional differences in target values. Europace. (2017) 19:775–83. 10.1093/europace/euw105 27247002

[23] Calzolari V De Mattia L Indiani S Crosato M Furlanetto A Licciardello C. In vitro validation of the lesion size index to predict lesion width and depth after irrigated radiofrequency ablation in a porcine model. JACC Clin Electrophysiol. (2017) 3:1126–35. 10.1016/j.jacep.2017.08.016 29759495

[24] Hartung WM Burton ME Deam AG Walter PF McTeague K Langberg JJ. Estimation of temperature during radiofrequency catheter ablation using impedance measurements. Pacing Clin Electrophysiol. (1995) 18:2017–21. 10.1111/j.1540-8159.1995.tb03862.x 8552515

[25] Harvey M Kim YN Sousa J el-Atassi R Morady F Calkins H. Impedance monitoring during radiofrequency catheter ablation in humans pacing. Clin Electrophysiol. (1992) 15:22–7. 10.1111/j.1540-8159.1992.tb02897.x 1370996

[26] Segreti L De Simone A Schillaci V Bongiorni MG Pelargonio G Pandozi C. A novel local impedance algorithm to guide effective pulmonary vein isolation in atrial fibrillation patients: preliminary experience across different ablation sites from the CHARISMA pilot study. J Cardiovasc Electrophysiol. (2020) 31:2319–27. 10.1111/jce.14647 32613661

[27] Ikeda A Nakagawa H Lambert H Shah DC Fonck E Yulzari A. Relationship between catheter contact force and radiofrequency lesion size and incidence of steam pop in the beating canine heart: electrogram amplitude, impedance, and electrode temperature are poor predictors of electrode-tissue contact force and lesion size. Circ Arrhythm Electrophysiol. (2014) 7:1174–80. 10.1161/CIRCEP.113.001094 25381331

[28] Neuzil P Reddy VY Kautzner J Petru J Wichterle D Shah D. Electrical reconnection after pulmonary vein isolation is contingent on contact force during initial treatment: results from the EFFICAS I study. Circ Arrhythm Electrophysiol. (2013) 6:327–33. 10.1161/CIRCEP.113.000374 23515263

[29] Kautzner J Neuzil P Lambert H Peichl P Petru J Cihak R. EFFICAS II: optimization of catheter contact force improves outcome of pulmonary vein isolation for paroxysmal atrial fibrillation. Europace. (2015) 17:1229–35. 10.1093/europace/euv057 26041872 PMC4535556

[30] Chikata A Kato T Yaegashi T Sakagami S Kato C Saeki T. General anesthesia improves contact force and reduces gap formation in pulmonary vein isolation: a comparison with conscious sedation. Heart Vessels. (2017) 32:997–1005. 10.1007/s00380-017-0961-z 28260190

