# Supplementary material for: Characteristics of Very High-Power, Short-Duration Radiofrequency Applications
Source: Front Cardiovasc Med. 2022 Jul 13;9:941434. doi: 10.3389/fcvm.2022.941434 (PMC9326019; doi:10.3389/fcvm.2022.941434)
Supplement: Supplementary file 1 [file Data_Sheet_1.pdf]

Supplementary material

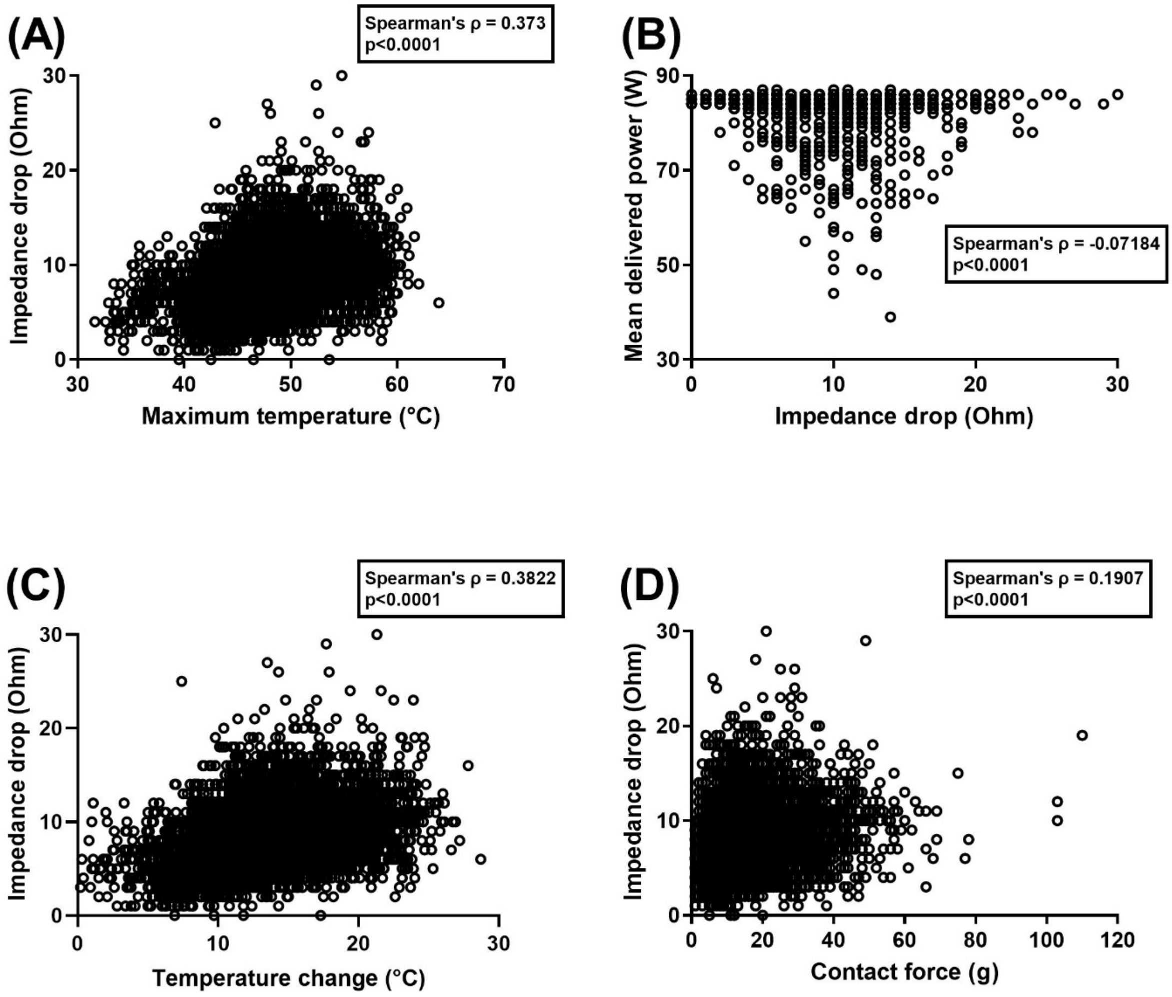

Figure S1A-1D: Scatter plot diagrams of the correlations between different parameters registered during radiofrequency applications. Panel **(A)**: correlation between the maximum temperature and impedance drop. Panel **(B)**: correlation between impedance drop and the mean delivered power. Panel **(C)**: correlation between temperature change and impedance drop. Panel **(D)**: correlation between contact force and impedance drop. Abbreviations: ρ = correlation coefficient.

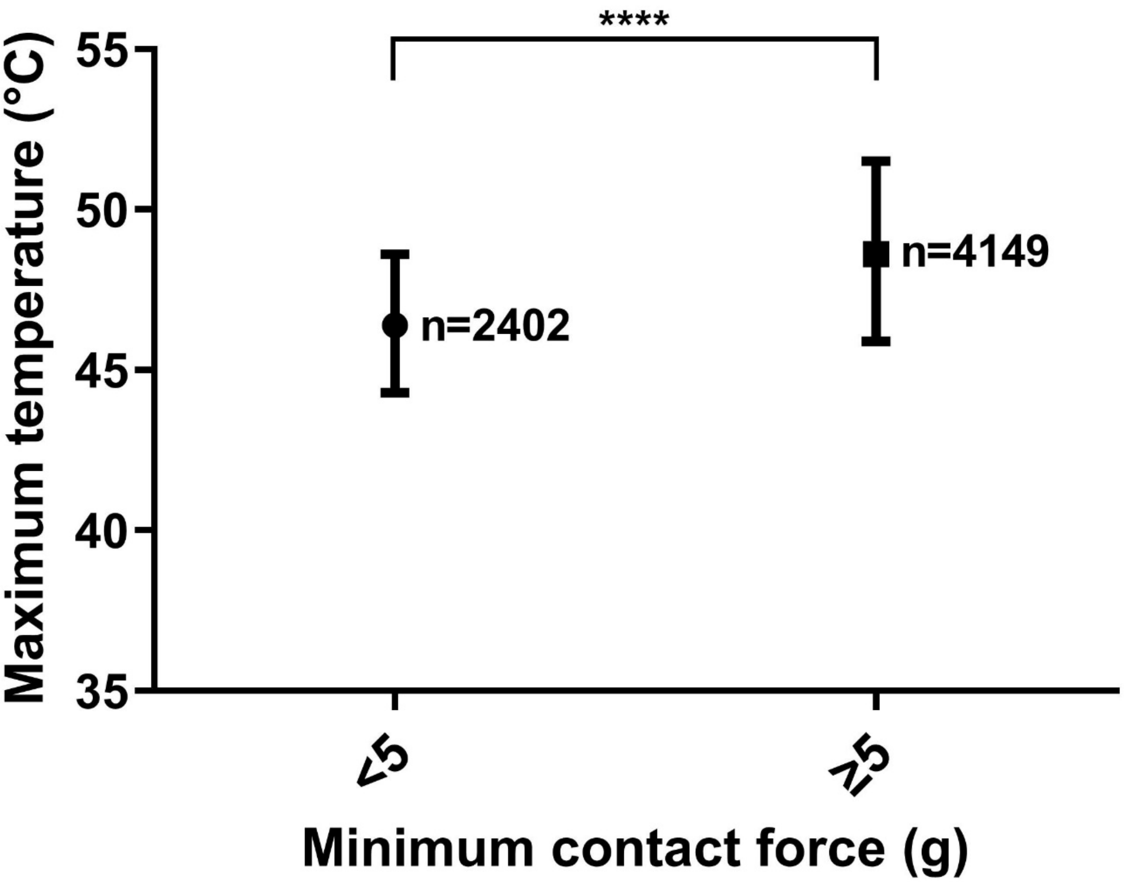

Figure S2: Difference in the maximum temperature between lesions created with a minimum CF less than 5 grams and those with a minimum CF greater than or equal to 5 grams. Abbreviations: **** = p<0.0001. Median and interquartile ranges.

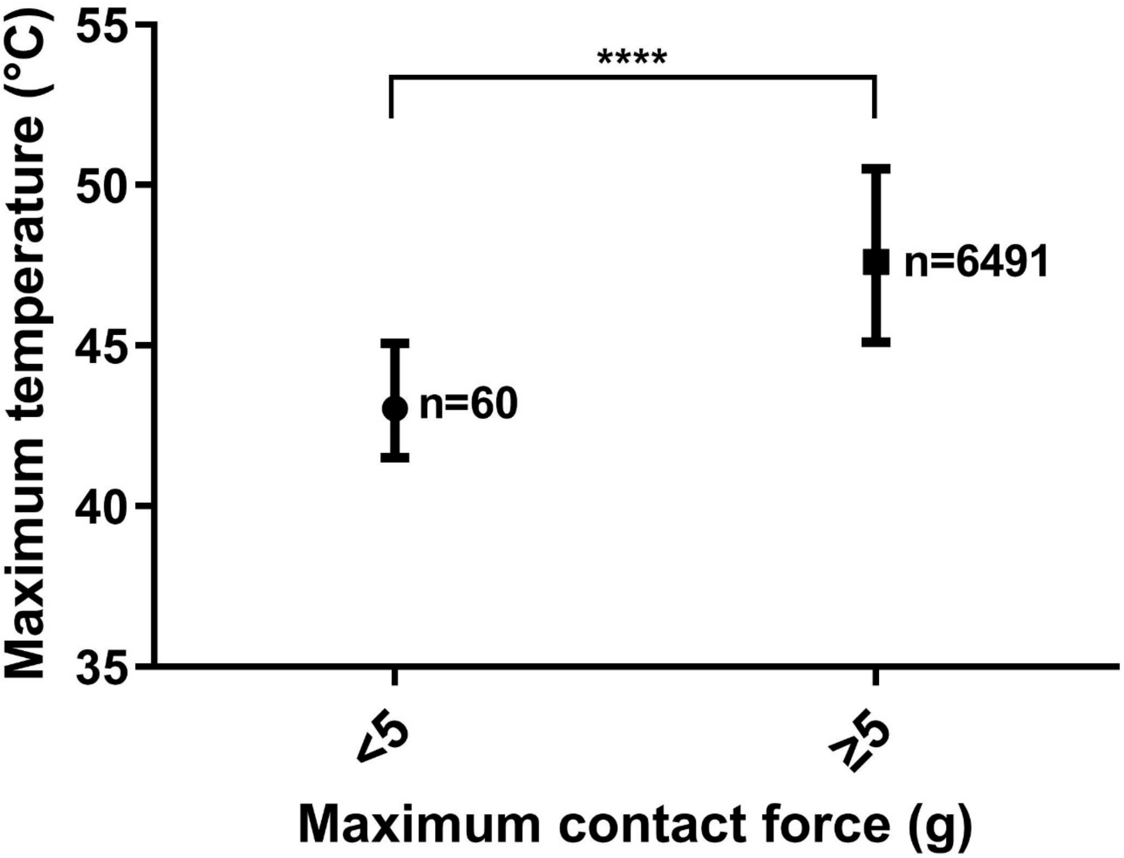

Figure S3: Difference in the maximum temperature between lesions created with a maximum CF less than 5 grams and those with a maximum CF greater than or equal to 5 grams. Abbreviations: **** = $p<0.0001$. Median and interquartile ranges.

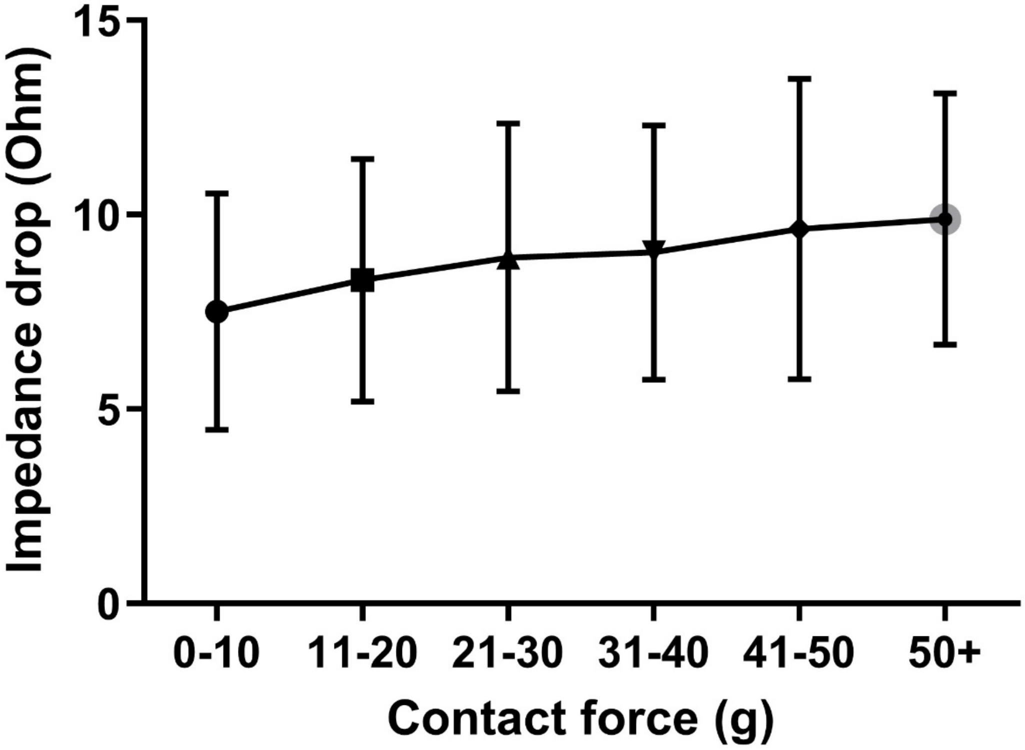

Figure S4: The association of impedance drop to the different contact force ranges. Increasing contact force values result in moderately higher impedance drop. Median and interquartile ranges.
